# Cholera

**Published:** 1884-08

**Authors:** 


### CHOLERA.

Cholera is now raging in several cities in France, and a large number of deaths are occurring daily. Dr. Koch, the German scientist, is now in Toulon, investigating the disease. He is of the opinion that it is genuine Asiatic cholera, and that it will spread everywhere. Whether this be true or not it behooves us to use all means at our disposal in preventing the disease, or in keeping our homes in a condition not favorable to the development of the disease. Our city authorities should see to it at once that Atlanta is placed in a good sanitary condition.

